# The Role of the AO Spine Primary Infection Classification in Evaluating Medical and Surgical Management: A Narrative Review

**DOI:** 10.1177/21925682261507650

**Published:** 2026-11-11

**Authors:** Barry Ting Sheen Kweh, Alexander R. Vaccaro, Gregory D. Schroeder, Mohamad M. Aly, Mohammad El-Sharkawi, Charlotte Dandurand, Jonathan Neuhoff, Brian A Karamian, Grace X. Xiong, Harvinder Singh Chhabra, Richard Bransford, Andrei Fernandes Joaquim, Jin Wee Tee

**Affiliations:** 1 National Trauma Research Institute, Spine and Neurotruma Division, Melbourne, Victoria, Australia; 2 Department of Neurosurgery, The Alfred Hospital, Melbourne, Victoria, Australia; 3 Department of Neurosurgery, Royal Melbourne Hospital, Parkville, Victoria, Melbourne; 4 Department of Orthopaedic Surgery, Rothman Institute, Thomas Jefferson University Hospital, Philadelphia, PA, USA; 5 Department of Neurosurgery, Prince Mohammed Bin Abdulaziz Hospital, Riyadh, Saudi Arabi; 6 Department of Orthopaedic and Trauma Surgery, Faculty of Medicine, 68796 Assiut University, El Fateh, Egypt; 7 Combined Neurosurgical and Orthopedic Spine Program, Vancouver General Hospital, University of British Columbia, Vancouver, BC, Canada; 8 Center of Spine Surgery and Neurosurgery, BG Unfallklinik Frankfurt am Main, Frankfurt, Germany; 9 Department of Orthopaedics, University of Utah, Salt Lake City, UT, USA; 10 Department of Orthopaedic Surgery, 6429 Stanford University, Redwood City, CA, USA; 11 309539 Sri Balaji Action Medical Institute, New Delhi, India; 12 Harborview Medical Center, Department of Orthopaedic Surgery, 7284 University of Washington, Seattle, WA, USA; 13 Neurosurgery Division, Department of Neurology, State University of Campinas, Campinas-Sao Paulo, Brazil; 14 Central Clinical School, Faculty of Medicine, Nursing and Health Sciences, Monash University, Melbourne, Victoria, Australia

**Keywords:** Infection, Osteomyelitis, Discitis, Epidural Abscess, Decompression

## Abstract

**Study Design:**

Narrative review.

**Objectives:**

To clarify definitions and classifications regarding the diagnosis, medical managementand role of spinal surgery in the treatment of primary pyogenic spinal infections.

**Methods:**

A comprehensive review of the MEDLINE, OVID, Embase, Google Scholar and Cochrane Database of Systematic reviews was conducted to characterize historical means of diagnosing spondylodiscitis and explore current controversies in the management of primary pyogenic spinal infections. This includes evaluating the role of aggressive early surgical intervention versus conservative maximal medical management. The AO Spine Primary Infection Classification System is introduced and framed against this historical backdrop.

**Results:**

Surgical intervention in the treatment of pyogenic spinal infection is traditionally reserved for cases of neurological deficit, mechanical instability, or to facilitate microbiological diagnosis. Contemporary studies contend that early surgical debridement leads to superior source control and improved functional as well as neurological outcomes. We note that the majority of these studies are retrospective in nature and that there is a consistent lack of a clear definition of spondylodiscitis or stage of pyogenic primary spinal infection, variation in antibiotic regimens and lack of clear definitions as to what constitutes early or late surgery.

**Conclusions:**

Patients who suffer from pyogenic primary spinal infections present at different time courses along their illness and therefore existing studies examining early or late surgical interventions are confounded by this heterogeneity. Existing studies remain disparate due to the absence of a reliable classification. The AO Spine Primary Infection Classification System presents a universal and standardized means of communicating severity of spinal infections.

## Introduction

Primary pyogenic spinal infection constitutes 2-7% of all musculoskeletal infections and exhibits a bimodal distribution, with incidence peaking at 20 years of age before recurring again between 50-70 years of age.^1^ The incidence of spondylodiscitis and epidural abscess mirrors the escalating prevalence of high-risk comorbidities in the setting of a burgeoning elderly population.^2^ This abrupt rise has been estimated to be as high as 44% within the past decade.^2,3^ Traditional surgical tenets would suggest surgical decompression and its attendant risks should be avoided unless there is neurological deficit, gross instability or microbiological diagnosis is required to facilitate targeted anti-microbial therapy.^4^

However, Thavarajasingam et al in their meta-analysis noted that early surgical decompression provides a morbidity and mortality benefit.^5^ Proponents of early surgical decompression posit that source control, reduced risk of delayed neurological deficit and definitive microbiological diagnosis represent formidable advantages of surgery. Indeed, Croes et al specifically contended that without surgical debridement of bacteria, resultant osteoblast activity and bone mineralization remain impaired.^6^ Intriguingly, this extends to the idea that early surgery may even be more effective in reducing mortality when performed in cases of spondylodiscitis without any epidural abscess formation .^5^ This remains a true area of uncertainty and controversy at the multidisciplinary interface between antimicrobial stewards and spinal surgeons, with a vast array of medical and surgical factors demanding consideration. Given the existing inconsistencies in diagnosis, assessment and management of these patients, we propose a rational method of classifying these patients on index presentation utilizing the AO Spine Primary Infection Classification System. This system has recently undergone international and internal validation with satisfactory inter-rater and intra-rater reliability, and is designed to accurately and consistently describe infections with the eventual future aim of predicting neurological outcomes and generating treatment algorithms.^7^

## Methods

A literature review was conducted to identify the classification systems used by studies examining outcomes in patients with primary pyogenic spinal infections. We defined primary pyogenic spinal infection as a bacterial infection manifesting as spondylodiscitis with or without an epidural abscess component that occurs spontaneously in the absence of any prior spinal surgery. The aim was to determine if there existed reliable and validated systems in the classification of these infections. Two independent authors (BTSK and JWT) searched the MEDLINE, OVID, Embase, Google Scholar and Cochrane Database of Systematic reviews from date of inception to June 2026 with the search terms ‘spinal infection’ AND ‘classification’ OR ‘system’. Original cohort studies, literature reviews and systematic reviews were included. Exclusion criteria included secondary or atypical infections. The themes and controversies generated form the basis for this literature review, which introduces the AO Spine Primary Infection Classification System and its potential utility in providing a universal language for classifying cases or primary pyogenic spinal infection.

### The AO Spine Primary Infection Classification System

The AO Spine Primary Infection Classification System (Figure 1) simplifies existing classification systems into a universal language. The regions of the spine are divided into Type A (Lumbar), Type B (Thoracic) and Type C (Cervical) with this progression reflecting that there is increasing risk of greater neurological deficit as primary pyogenic infections ascend cranially. Across the spine, the A0/B0/C0 patterns (Figure 2) are classified as extraspinal infections whilst the A1/B1/C1 (Figure 3) patterns represent cases of osteomyelitis with or without discitis in the absence of canal involvement. This is followed by the A2/B2/C2 patterns in which there is canal involvement without neural compression (Figure 4). Finally, the most severe form is the A3/B3/C3 subtype in which there is canal involvement with neural compression (Figure 5). For Types 1-3, addition of a “+” signifies bony destruction which alerts clinicians to the possibility of instability (Figure 5). If infection spans multiple levels or occurs at a junctional level, then the most cranial level affected is utilized to classify the infection. That is to say that an infection at C7/T1 which causes neural compression would be classified as C7 Type C3.

**Figure 1.**
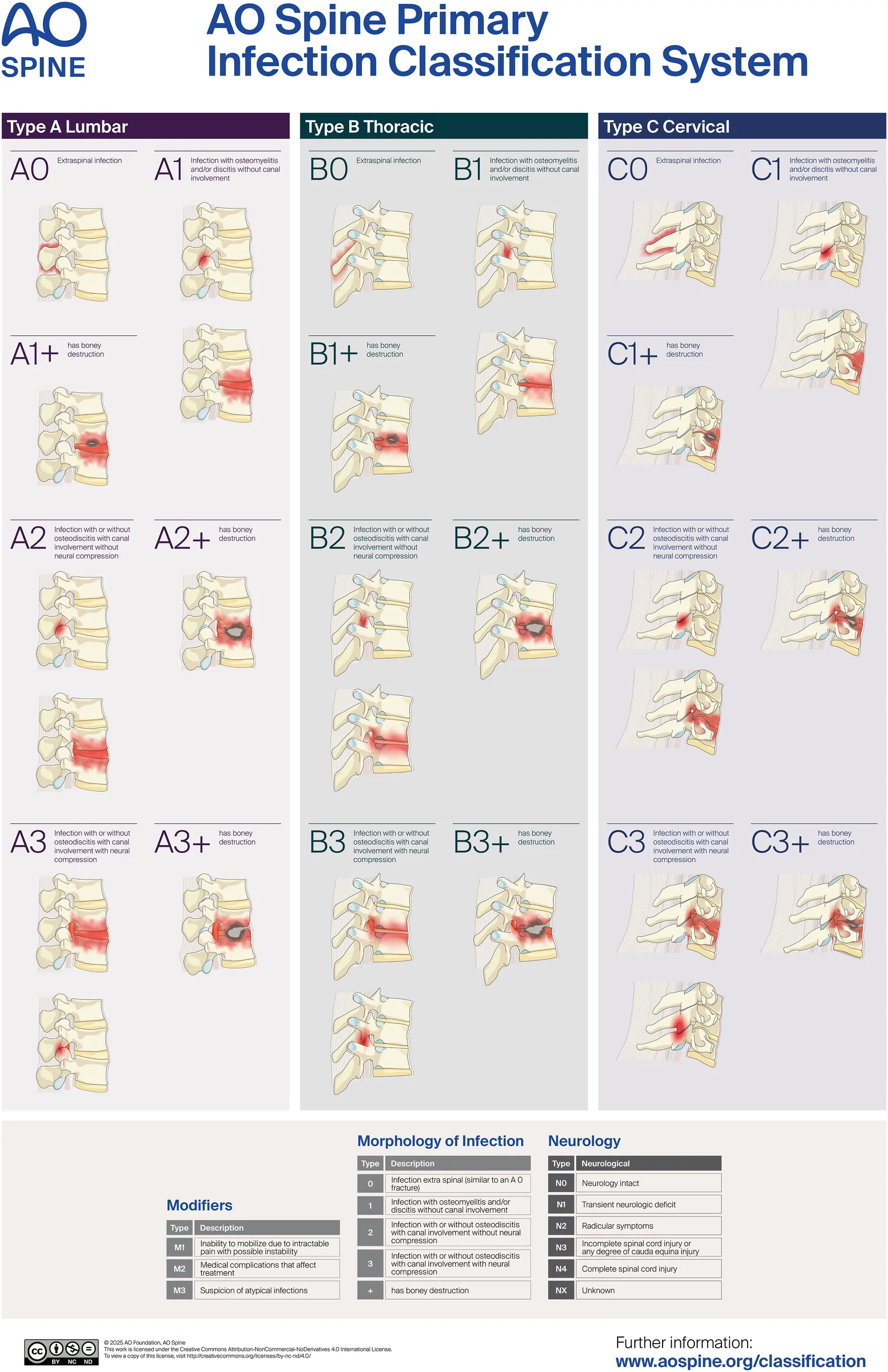
AO Spine Primary Infection Classification System with the separation into lumbar (Type A), thoracic (Type B) and cervical (Type C) categories. Escalating types of infection are depicted with increasing degrees of potential for neurological compromise and mechanical instability

**Figure 2.**
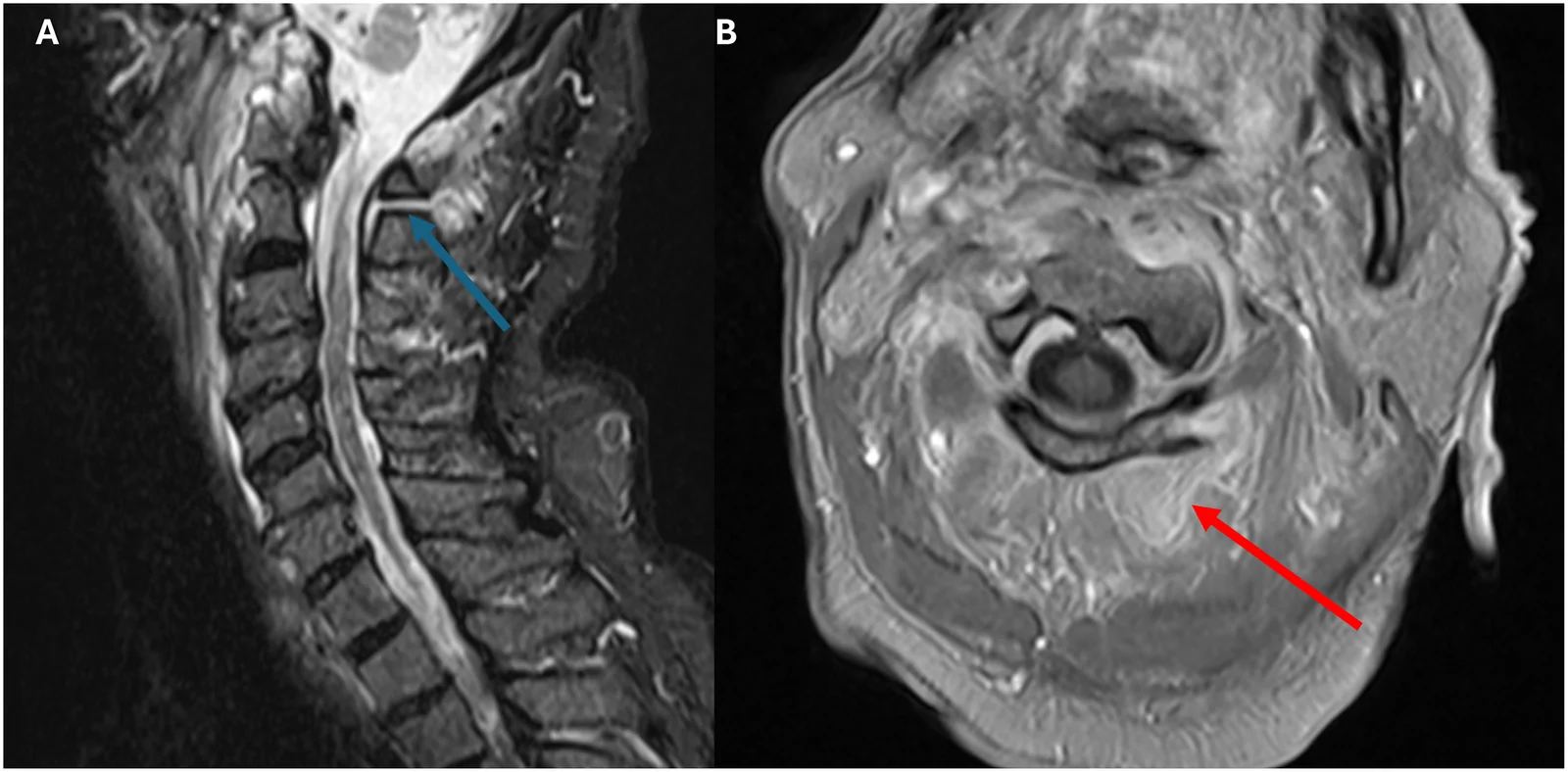
Type C0 infection demonstrated on short tau inversion recovery (STIR) sagittal (A) images and T1 post contrast axial (B) images. There is dorsal STIR signal change at the level of C1/2 with fluid within the facet joint (blue arrow) as well as left paraspinal musculature enhancement (red arrow) but no canal involvement or neural compression

**Figure 3.**
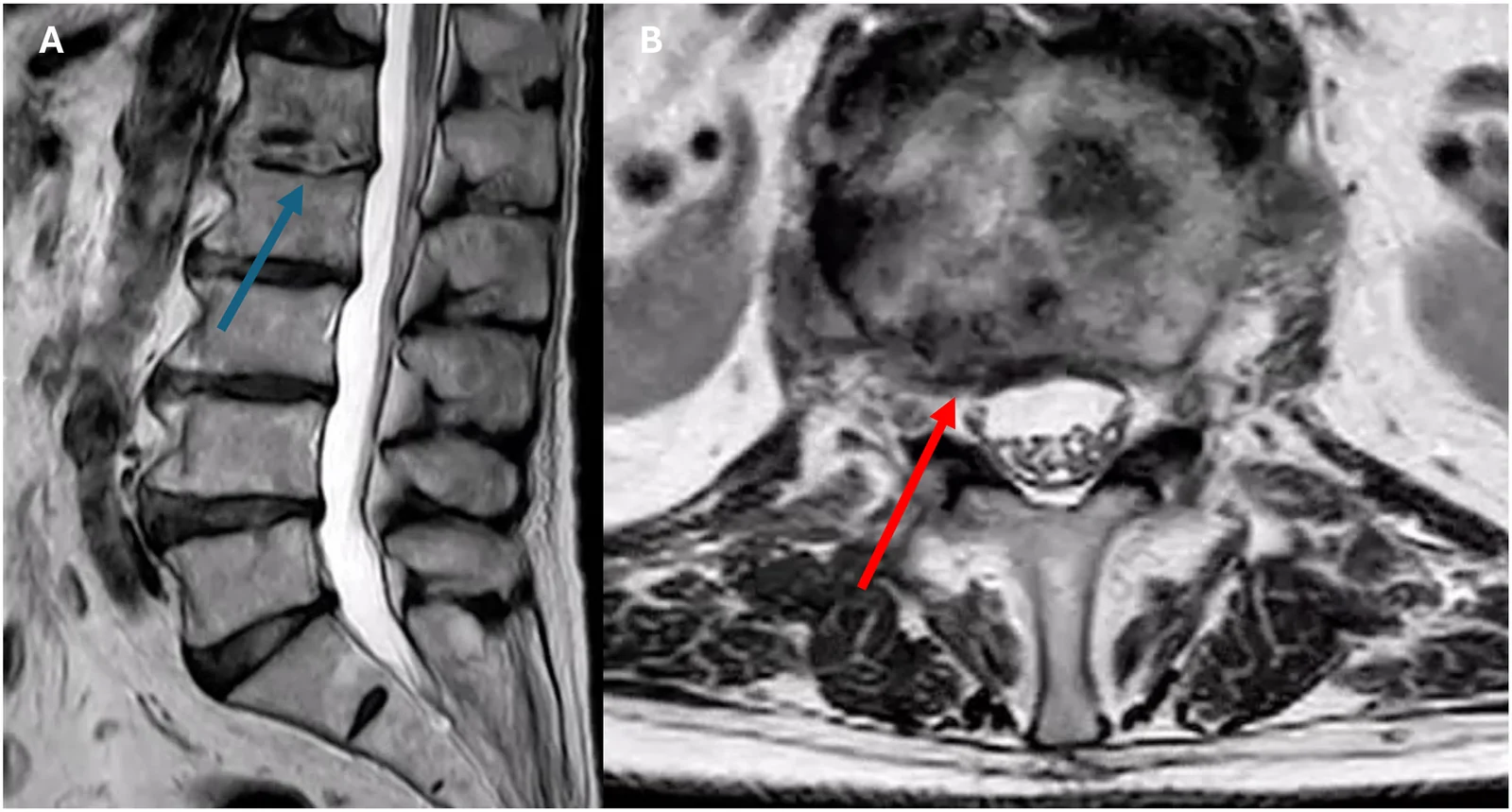
Type A1 infection demonstrated on T2 weighted magnetic resonance imaging sagittal (A) and axial (B) with L1/2 discitis and T2 hyperintensity within the disc space (blue arrow) without epidural extension or canal stenosis. Notably, there is pre-existing right sided disc degenerative disease with a disc prolapse (red arrow) which is discrete and, even if it caused canal narrowing, would not upgrade the infection to a higher category alone

**Figure 4.**
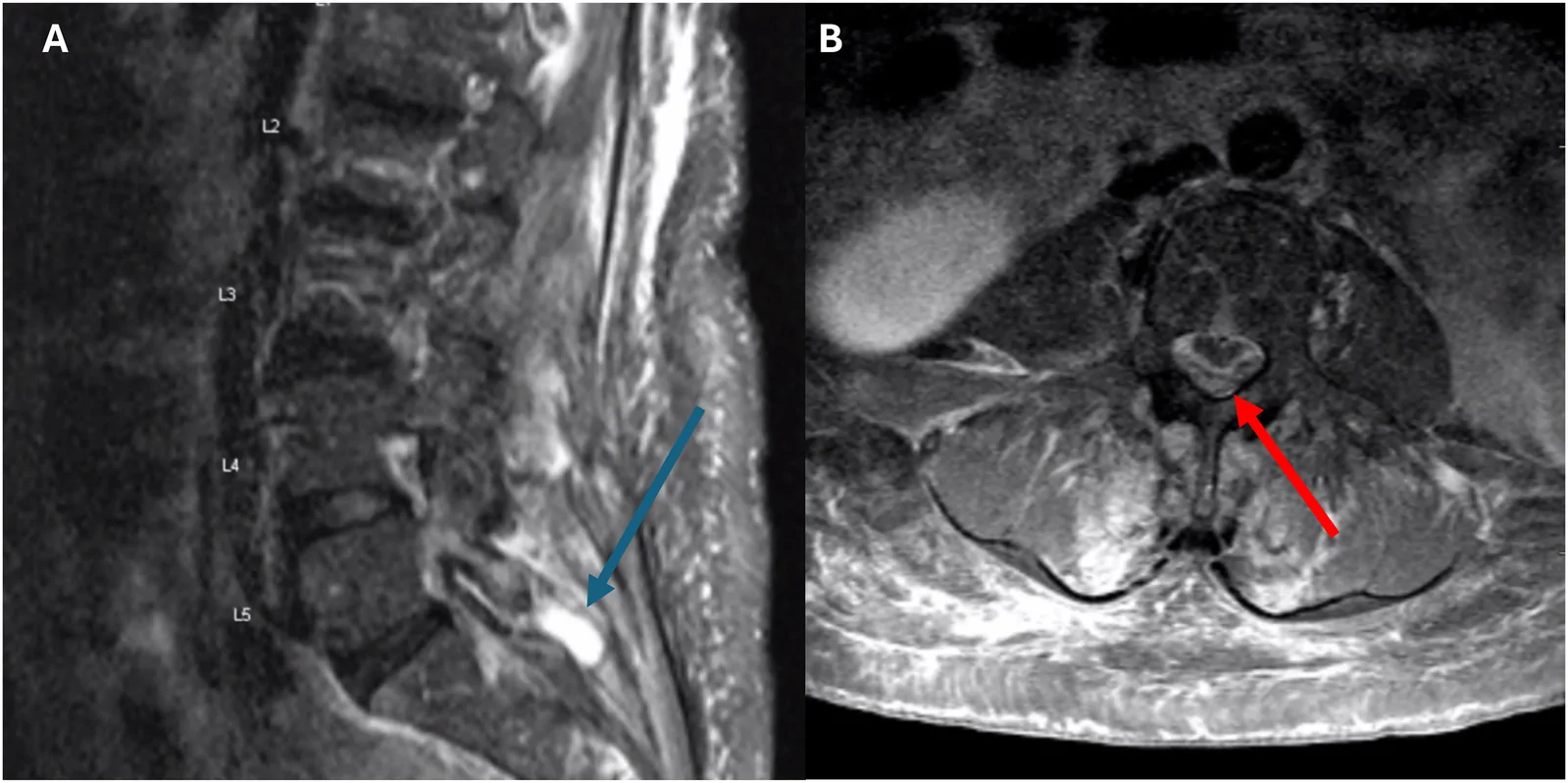
Type A2 infection demonstrated on short tau inversion recovery (STIR) sagittal (A) and axial (B) sequences. There is an extra-spinal paraspinal collection (blue arrow) which would be classified as a Type A0 infection alone, but axial imaging reveals a thin dorsal collection (red arrow) without canal stenosis which is consistent with a Type A2 infection

**Figure 5.**
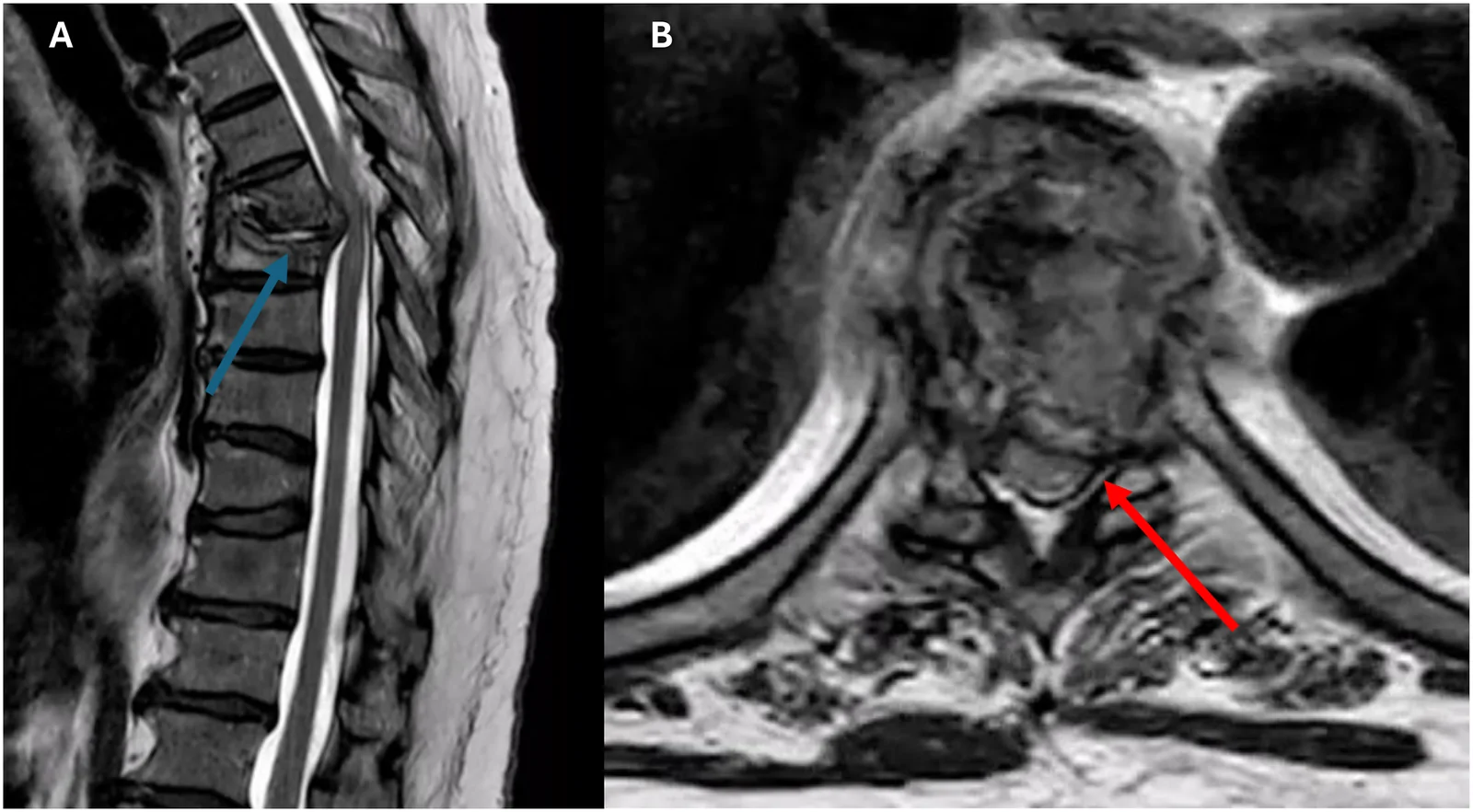
Type B3+ infection illustrated on T2 weighted magnetic resonance imaging sagittal (A) and axial (B) imaging confirms a severe discitis and vertebral body bony destruction with local kyphosis (blue arrow). There is ventral left sided epidural extension and severe cord compression (red arrow) and resultant cord signal change. This is the most severe form of infection and likely requires surgical decompression and fixation

Similar to the AO Spine trauma classification systems, there are important modifiers which describe crucial factors that may alter surgical decision making.^8-15^ This includes M1 signifying inability to mobilize due to intractable pain with possible instability, M2 heralding significant medical complications that affect treatment and M3 raising suspicion of atypical infections. These atypical infections include tuberculosis and fungal infections which require vastly different medical and surgical approaches compared to the more common pyogenic primary spinal infections. The rationale for the M2 modifier is based upon the astute study of Kilinc et al who contended that not only is infection morphology important, but so too the frailty status of the patient as measured by the Charlson Comorbidity Index (CCI).^16^ Vettivel et al supported this finding, reporting that a higher modified frailty index was associated with an increased risk of mortality.^17^ Indeed, Joerger et al found that diabetes, or even a history of steroid use, conferred a greater risk of mortality.^18^

A separate designation of neurological status also assists physicians in conveying with each other the severity of deficit caused by the infection. The N0 category denotes a neurologically intact patient and, as supported by Duarte et al, these are the patients in whom conservative management is most likely to be reasonable in the absence of spinal instability (absence of M1 modifier).^19^ In a graded fashion, N1 represents a transient neurological deficit whilst N2 is consistent with ongoing radicular symptoms. Finally, N3 heralds incomplete spinal cord injury or any degree of cauda equina injury with this escalating to N4 being complete spinal cord injury. Surgical intervention is recommended for those patients with canal involvement consistent with the observations of Kehrer et al who found that mortality was greatest in those patients with epidural abscess formation and thus neurological deficit.^20^

There are a number of nuances within the AO Spine Primary Infection Classification System that demand deliberate consideration. For example, a cervical rather than lumbar epidural collection carries the potential to cause tetraplegia as Shweikeh et al noted.^21^ Hence, this may require earlier surgical decompression given the potential for devastating neurological sequelae.^21^ Gentile et al supported this tenet in that cervical and thoracic cases of epidural abscess were more likely to present with neurological deficit.^22^ As such, clinicians should remain particularly vigilant in cases of Type B and Type C infection. Importantly, continuous re-evaluation of patients should be conducted given that infection may progress in terms of the degree of regional spinal involvement, such as an ascending or descending infection as well as the capacity for infection to progress despite conservative management and to evolve rapidly into A3/B3/C3 patterns. Indeed, Lemaignen et al found that neurological deterioration may occur in 30% of patients, but that functional outcome may remain acceptable with early intervention.^23^ This holds true even where surgery is slightly delayed but still performed, as Buschmann et al noted, with the surgical treatment group retaining an acceptable 1-year mortality rate at long-term follow-up together with favourable quality of life outcome measures including the visual analogue scale and Oswestry Disability Index (ODI).^24^

### Synthesis of Available Evidence

#### Flaws in Existing Spinal Infection Classification Systems

The AO Spine Primary Infection Classification System represents an advancement in clinician understanding and ability to communicate the severity of primary spinal infections in a hierarchical fashion. Various historical classification systems have been proposed but none have been consistently utilized. Pola et al offered a clinico-radiological system of pyogenic spondylitis based upon a series of 250 patients.^25^ In this classification, three classes were identified depending on whether there was bony destruction or segmental instability, epidural abscess, or neurological impairment.^25^ These appear to have been identified as crucial determining factors without any attempt at rationalizing a hierarchy of escalating severity to guide surgical decision-making, limit complications and costs, and avoid overtreatment.

Pluemer et al advanced this work by comparing three objective scoring systems for spondylodiscitis. These scoring systems were the spinal infection treatment evaluation score (SITE), spine instability neoplastic score (SINS), and spinal instability spondylodiscitis score (SISS).^26^ The SITE score represents an attempt at categorizing infection and subsequently guiding clinicians as to whether surgical intervention is required based upon 5 key domains: neurology, location, radiology, pain and host comorbidities.^26^ In comparison, the SISS utilizes location, bone lesion and extent of vertebral body involvement, spinal alignment and mechanical pain, whilst the SINS was derived and validated for neoplastic rather than infective disease and is therefore only indirectly applicable in this setting. The authors found the SITE score showed the greatest promise with regard to inter-rater and intra-rater reliability, but still requires further external validation.^26^

When compared and assessed on external performance by Xiong et al, the unique combination of variables employed by the SITE score proved more useful in predicting outcomes compared to the SISS score.^27^ This was tested by comparing clinician decision-making, whether operative or non-operative, on 213 cases utilizing the scoring systems, with the real-world treatment delivered as the gold standard.^27^ However, it was clearly noted that these systems still fail to provide a logical means of classifying spinal infection that can be utilized in future studies to risk stratify outcomes. Whilst each of the aforementioned schemes may be useful in certain circumstances, there is still a need for a common language when describing infections in future research. The AO Spine Primary Infection Classification System provides a stepwise stratification of infection that forms a global language as a vehicle for more accurate communication and research.

### Simplifying the Stages of Spinal Infection

The heterogeneity and lack of a standardized classification system for spinal infections likely stem from the fact that there is a variable timepoint from which infections are detected. As such, any combination of medical or surgical treatment is judged for its effectiveness in patients who are at different stages of pyogenic spinal infection. This is compounded by a lack of clarity in the literature as to which classification system, if any, is applied when studies are conducted. Fortunately, the AO Spine Primary Infection Classification System assists in clarifying this by providing a logical hierarchy in which studies can document the current stage of infection, ranging from Type 0 to Type 3, which is specifically being investigated when comparing interventions.

For instance, some patients may be diagnosed very early in the course of their disease when only mild back pain is noted but the entire panel of their biochemistry and neurological examination remains unremarkable. The earliest example of radiological detection is indeterminate MRI disc hyperintensity or faint enhancement, which may be confirmed as significant with advanced nuclear imaging. Indeed, Maamari et al found that, of all imaging modalities, 18F-FDG PET/CT demonstrated superior sensitivity (93%) and specificity (80%) compared with MRI (90% and 72% respectively).^28^ Such a case, where appropriate medical management is instituted early, is likely to result in antimicrobial therapy being more effective than a patient in whom there is already a thin non-compressive epidural phlegmon at multiple spinal levels who remains neurologically intact but is still further along the pathological course than the former patient. The latter is readily classified as a Type 2 spinal infection pattern using the AO Spine Primary Infection Classification System.

From this arises the current controversy regarding exact definition of an early or late presentation. Epstein et al argued that surgery is typically categorized as early if performed within 24 hours and late thereafter.^29^ Ghobrial et al subscribed to the same definition in differentiating early from late surgery, but in their retrospective study found that those undergoing delayed surgery were significantly older, which is likely again a result of surgeons preferencing medical management in the more comorbid patients initially.^30^ This is further supported by the observation of Farber et al who analyzed 10,150 patients and found that the rate of postoperative infection and paraplegia was highest with early surgery (p<0.0001), precisely because these patients were likely more acutely unwell or suffering from a neurological deficit.^31^ It is evident that there is a lack of consensus regarding the diagnosis and classification of spinal infections.

This is exemplified when Kramer et al uncovered significant international variability in the treatment of spondylodiscitis, although most neurosurgeons favoured conservative management.^32^ Superficially, the study appears robust in its methodological design in which patients were divided into three groups: early surgery (within three days of admission), delayed surgery (after three days of admission) and conservative therapy.^32^ Yet the conclusion that delayed surgery, optimally between 10-14 days after admission, resulted in lower 30-day mortality (4.1%) compared to early surgery (27.9%) is confounded by the fact that days since admission is not as relevant as length of time since the disease process started.^32^ These findings are counterintuitive, in that patients with a neurological deficit would preferentially undergo early surgery; the delayed surgery cohort therefore likely comprised patients who were initially well but subsequently experienced infection progression. These examples of confounding by indication and lead-time bias would be minimized by the use of the AO Spine Primary Infection Classification System. This allows clinicians to document accurately and universally the stage of infection upon diagnosis and at every point of progression or regression, rather than relying on arbitrary metrics such as days since admission. It is envisioned the role of the classification would be akin to that of the American Spinal Injury Association (ASIA) in spine trauma patients, for whom the neurological status is initially documented and the degree of recovery within this grading system can then be utilized to measure outcomes.

### Early Surgical Intervention versus Conservative Management

It is also evident in the literature that the degree of operative decompression and source control performed for early spinal infection is variable and inconsistent. Using the AO Spine system, Type 3 infections are the most severe with neural compression whereas Type 2 infections are characterized by canal involvement without neural compression.^33^ Type 2 infection is characterized by canal involvement without radiological evidence of neural compression. Neurological status should be recorded separately using the N grade because morphology alone does not determine the neurological examination. There is minimal contention that patients with concomitant neurological compromise require surgical intervention. Within the Type 2 category itself though, there is a vast difference between the possible gradated options of washout of muscular abscess and percutaneous debridement performed by Uchida et al, washout of epidural phlegmon alone and flavectomy suggested by Lee et al or Lener et al, or the most aggressive surgical intervention represented by evacuation of a liquid abscess component followed by curettage of all phlegmon from the thecal sac as well as disc space and affected vertebral body by Mann et al^1,34-37^

It is also rarely stated if surgeons concurrently performed washout of sites of non-contiguous disease simultaneously if an operation was being performed at a single compressive site. As such, these treatment options are often all amalgamated together by studies when each is specifically targeted at a different stage of the AO Spine Primary Infection Classification System. Type 0 para-muscular infections are generally most amenable to percutaneous drainage and there is rarely a role in including these infections in most comparative studies attempting to evaluate medical versus surgical management.^38^ Similarly, Type 1 infections, comprising osteomyelitis with or without discitis, are usually amenable to medical management.^39^ The classification is currently descriptive but its intentional hierarchical nature will ideally be evaluated in future studies.

Surgical evacuation of pyogenic primary spinal infections is performed for decompression of neural elements, but also to reduce bacterial load at that site. An additional potential advantage is the reduced risk of venous thrombophlebitis leading to a congestive myelopathy, with the thoracic cord at particular risk of developing this devastating non-compressive neurological deficit. It is notable that none of the existing studies clearly defined what surgical intervention actually entailed in a heterogeneous group of patients or examined more complex multi-factorial benefits of surgery (Table 1).

**Table 1.** Literature review of studies comparing medical versus surgical management of spondylodiscitis

Authors & Year	Study Type	n	Overall Finding	Study Findings
Patel et al, 2014^40^	Retrospective Cohort	128	Surgery	Early surgery rather than conservative management results in improved neurological outcomes
Wang et al, 2019^41^	Meta-Analysis	808	Equipoise	Similar neurological outcomes with surgery or conservative management Recommend individual case by case basis
Farber et al, 2017^31^	Retrospective Cohort	10,150	Surgery	Reduced mortality and sepsis with shorter length of stay with operative rather than medical management
Tsai et al, 2017^42^	Retrospective Cohort	90	Surgery	Reduced hospital stay and improved functional outcomes with surgery compared to conservative management
Canoui et al, 2019^43^	Retrospective Cohort	90	Equipoise	Similar improvement in neurological deficit and reduction in pain between operative and conservative management
Alas et al, 2020^44^	Retrospective Cohort	116	Surgery	Reduced 12 month mortality with operative rather than conservative management Higher modified frailty index (MFI) scores associated with increased mortality
Hohenberger et al, 2022^37^	Retrospective Cohort	54	Surgery	Early rather than late dorsal fusion results in shorter hospital stay
Thavarajasingam et al, 2023^5^	Meta-Analysis	10,954	Surgery	Reduced relapse/failure, mortality and length of stay in surgery compared to conservative management
Buschmann et al, 2025^24^	Retrospective Cohort	103	Surgery	Reduced 12 month mortality with operative rather than conservative management Similar quality of life measured with the Parker Mobility Score
Neuhoff et al, 2025^45^	Retrospective Cohort	190	Surgery	Reduced mortality and hospital stay with operative rather than conservative management

Thavarajasingam et al conceded this after their systematic review of 31 studies found that multiple definitions for failure of treatment were utilized including any failure of initial medical management leading to surgical intervention or failure of surgical intervention requiring repeat surgery.^5^ Both of these scenarios rely upon the treating clinician following institution-dependent management algorithms. Use of the standardized AO Spine Primary Infection Classification System stratifies primary pyogenic spinal infections and facilitates accurate tracking of clinical status in a universal fashion.

Most surgeons utilize a combination of clinical, biochemical as well as radiological means of monitoring the trajectory of a patient with infective pathologies. On one hand, a patient whose pain is clinically improving, remains afebrile and demonstrates normalizing inflammatory biochemistry markers is likely to improve irrespective of surgical intervention. Enrolling this patient into either an early surgical intervention arm or maximal medical management cohort falsely inflates the treatment success rate of that arm. At the opposite end of the spectrum is a patient who is septic with deteriorating neurological function and an obvious compressive collection that the majority of surgeons would agree requires urgent decompression. Arguably these two patients should be excluded from retrospective or prospective arms of treatment, but the lack of a quantifiable classification system or treatment algorithm results in the inclusion of these patients. The true area of equipoise is the cohort of patients who are neurologically intact with static biochemistry markers and occasional intermittent fevers in whom radiologically there is stability or perhaps only marginal interval change. It is thus unclear whether immediate surgical intervention or close observation results in favourable outcomes.

### Flawed Existing Evidence due to Heterogeneous Classification

Current studies are flawed in their methodology due to a heterogeneous cohort of patients of varying severity of infection. A means of avoiding the inadvertent inclusion of disparate populations within the same cohort would be the use of a standardized classification system. Inspecting the individual studies such as that by Patel et al reveals the potential for selection bias.^40^ This retrospective 128-patient study of patients with bacterial spinal epidural abscesses concluded that early surgery resulted in improved outcomes, but it is likely that only patients deemed fit for surgery underwent the more aggressive intervention.^40^ Likewise, Alas et al found that operative patients were more likely to be younger and less frail thereby cautioning that early surgical intervention may only be beneficial in an appropriately robust patient (Table 2).^44^

**Table 2.** Outcome-specific findings and methodological quality of studies comparing surgical versus conservative/medical management of spinal infection

Study (Design, N	Diagnostic Criteria or Imaging	Indications for Surgery	Neurological Deficits	Type of Procedure	Primary Outcome Measure(s)	Outcome-Specific Findings (Favours)	Methodological Quality/Adjustment for Confounding
Patel et al, 2014^40^	MRI and/or intraoperative findings	Bacterial spinal epidural abscess	Surgical patients had worse baseline ASIA motor scores (80.3 vs 97.9).	Laminectomy, laminotomies, ACDF, corpectomy an posterior spinal instrumented fusion	ASIA Score	Favours surgery for neurological recovery (+9.5 vs −5.9, unadjusted).	Retrospective, single-centre, non-randomized. No adjustment for baseline severity. Groups unbalanced (surgical patients more impaired).
Wang et al, 2019^41^	MRI/CT-myelogram	Individual surgical choice according to included studies	80% overall had deficit. Trend towards more deficits in poor-outcome surgical patients.	Laminectomy/decompression ± fusion; some percutaneous drainage.	Neurological outcome (good vs poor) at ≥6 months.	Similar outcomes surgery vs conservative (OR 1.01, p=0.98). Delayed surgery (>7d) worse than early surgery (OR 0.10).	Meta-analysis of retrospective series only. High heterogeneity.
Farber et al, 2017^31^	Spontaneous spinal abscess (records retrieved by ICD-9 code)	Individual surgeon preference	Paralysis 18.3% overall; more common with early surgery (20.5%) than medical care (9.4%), p<0.0001.	Laminectomy/decompression, instrumentation, <48h or ≥48h from admission.	In-hospital mortality, complications, length of stay, charges.	Adjusted analysis favors early over delayed surgery for mortality (OR 1.43), sepsis (OR 1.74), stay and charges (all p<0.0001).	Large administrative database study. Adjusted for patient/hospital covariates, but not for abscess severity or neurological status.
Tsai et al, 2017^31^	MRI	Early-stage infection, no deficit; allocation by patient preference.	None – absence of deficit was an inclusion criterion (0% both groups).	Anterior and/or posterior debridement and fusion, per surgeon preference.	Antibiotic duration, hospital stay, kyphosis correction, 12-month ODI/VAS.	Favours surgery: shorter antibiotics (p<0.001) and stay (p=0.0001), better kyphosis correction, ODI and VAS at 12 months.	Retrospective, single-centre, preference-based allocation. Unadjusted. Sickest patients excluded; Only 68% retained at follow-up.
Canoui et al, 2019^43^	MRI	Neurological deficit, instability, persistent infection, or worsening pain.	32% overall; 75% of surgical vs 13% of medical patients (p<0.001) – an indication for surgery.	Drainage/debridement in all (100%); laminectomy 61%; instrumentation 71%.	Neurological/pain improvement at 4 months; recurrence/death at 12 months.	After propensity-score adjustment, NO difference for neurological improvement (aOR 0.73) or pain (aOR 0.21, borderline). No difference in mortality or recurrence. Surgery equally effective, not superior.	Retrospective, single-centre. Propensity-score weighting and adjusted regression used – one of few adjusted analyses here. Small surgical group (n=28).
Alas et al, 2020^44^	MRI	Failed nonoperative therapy with sepsis/pain, instability, or neurological compromise.	Not reported. Cord/thecal sac compression more common in operative patients (28% vs 8%, p=0.004).	Posterior-only (49%), combined anterior-posterior (12%), or anterior-only (5%).	30-day and 1-year mortality; recurrence.	Favours surgery for 30-day mortality (2.3% vs 17.8%, p=0.016, unadjusted) and stay (p=0.002). No difference at 1 year or in recurrence.	Retrospective, single-centre. Operative patients significantly younger and less frail – confounding by indication not adjusted for in the mortality comparison.
Hohenberger et al, 2022^37^	MRI	Individual surgeon preference	52% overall; far more common in surgical groups (71–78%) than conservative (0%).	Open instrumented dorsal fusion (early or late), or decompression alone (no fusion).	Length of hospital stay; secondary – CRP, VAS pain, neurological status, complication.	Favours early over late fusion for hospital stay (33 vs 57 days, p=0.016). No significant difference in complications or pain relief.	Retrospective, single-centre, small subgroups (n=7–18). Groups not comparable at baseline.
Thavarajasingam et al, 2023^5^	MRI – pooled from studies	Individual surgeon preference	Not pooled as one figure from included studies.	Heterogeneous – laminectomy/decompression, debridement, abscess drainage ± fusion.	Mortality, relapse/failure, length of stay (pooled); neurological outcomes narrative.	Favours early surgery for mortality (RR 0.61, p<0.01), relapse/failure (RR 0.60, p<0.01), and stay (−7.75 days, p<0.01).	PRISMA/PROSPERO meta-analysis. Mostly moderate risk of bias (ROBINS-I). High heterogeneity. No RCTs. Authors flag likely selection bias, unadjusted.
Buschmann et al, 2025^24^	MRI	Individual surgeon preference	Not reported.	Radical debridement of affected levels with dorsal or ventral instrumentation as needed.	1-year mortality/survival; quality of life (EQ-5D-5L, ODI, Parker score) as secondary outcome.	Favours surgery for 1-year mortality (22% vs 40%, p=0.003) and treatment failure (19% vs 39%, p=0.032). No difference in quality of life.	Retrospective, single-centre. Unadjusted comparisons. Small sample with high loss to follow-up for quality-of-life outcomes.

ACDF = Anterior Cervical Discectomy and Fusion, ASIA = American Spinal Injury Association; CRP = C-reactive protein; ESR = erythrocyte sedimentation rate; JOA = Japanese Orthopaedic Association; ODI = Oswestry Disability Index; VAS = Visual Analogue Scale; OR = odds ratio; aOR = adjusted odds ratio; RR = relative risk; CI = confidence interval; mFI = modified frailty index; IPTW = inverse probability of treatment weighting; ROBINS-I = Risk Of Bias In Non-randomised Studies of Interventions; RCT = randomised controlled trial.

This exact area of equipoise was investigated by Tsai et al, who conducted a single-centre retrospective cohort study of patients in whom there was early stage infection without neurological deficit.^42^ Whilst the cohort of patients who underwent surgery followed by a course of post-operative antibiotics demonstrated reduced mean hospitalization periods and desirable kyphotic angle correction, infection control was deemed similar between the cohorts.^42,46-48^ Typical of studies in this field, neither the actual intention nor the operation performed was described in any meaningful detail.^42^ Verla et al performed a similar study, examining only patients with thoracolumbar junction discitis or osteomyelitis, a selection which inherently favours the operative cohort.^49^

Along a similar vein, Neuhoff et al conducted a multi-centre retrospective study of 392 patients comparing the ethos of treatment between two centres situated in the United Kingdom (UK) and Germany.^45^ In the UK, a conservative approach is consistently favored, in juxtaposition to the more operatively inclined trend observed in Germany.^45^ The authors concluded that the mortality rate was significantly higher in the conservative arm compared to the surgical arm (24.2% versus 4.2%, p<0.001).^45^ However, no significant difference was noted in infection relapse or neurological status at discharge. The international nature of this study is both a strength, in lending external validity, and a weakness, given that comparison of patients within the same centre rather than across centres would be more effective in eliminating bias.^45^ The cohorts are superficially comparable given the study conducted propensity matching with respect to age, pre-existing conditions and CRP levels.

However, these are perhaps the least important factors given an objective frailty index has been demonstrated to be more important in perioperative risk stratification.^47,50,51^ On the contrary, the absolute CRP level has not been demonstrated to be an independent predictor alone. A significant flaw in this study also remains the lack of long-term follow-up. A similar weakness is found in the study by Canouï et al who examined a pure cohort of pyogenic vertebral osteomyelitis and concluded medical management was equivalent to surgical management with respect to neurological deficit and recurrence of infection.^43^ These studies are interesting but all fallible in the absence of a clear means of classifying the cohort of patients actually being examined.

### Broader Inconsistencies

Furthermore, infectious disease physicians treat the same culprit organisms in a vast array of ways, ranging from dosing regimens and duration of antibiotics to the use of synergistic anti-microbials. Bernard et al attempted to resolve this conflict by comparing 6-week versus 12-week treatment of pyogenic vertebral osteomyelitis with antibiotics with the primary endpoint of cure at one year.^52^ Of the 359 patients apportioned to each treatment arm, the 6-week regimen was non-inferior to the 12-week regimen.^52^ Despite the study design, there is the potential for significant flaws including selection bias given patients were excluded if they failed to attend or were deceased. Rutges et al in their own systematic review proffered that an 8-week course of antibiotics may be utilized, demonstrating the individualized, patient-tailored approach often required in these cases.^53^ Furthermore, the study is not entirely reflective of daily clinical management, given follow-up visits usually involve tracking clinical and radiological progress before cessation of antibiotics and simply providing a general rule that high-risk patients who are immunocompromised or elderly should undergo an extended course of antibiotics is unhelpful.

Unfortunately, even larger meta-analyses such as that performed by Thavarajasingam et al consist of a large pool of varied patients without focusing specifically on the area of equipoise which triggers the most deliberation in physicians.^5^ This should be taken into consideration by readers when interpreting the conclusion that the pooled mortality was 8% for surgically managed patients versus 13% for patients initially treated conservatively.^5^ Furthermore, a 39% reduction in mortality rate with early operative intervention with a resultant 7.75-day reduction in length of hospital stay may not necessarily be meaningful if the aforementioned patients were included, which falsely elevates response rates.^5^ Bigdon et al have also critiqued this study and concurred that the results should be applied only provisionally, and higher quality studies are required.^5,54^ As testament to this, the diametrically opposite conclusion has been drawn by Wang et al in their systematic review and meta-analysis of 808 patients collected from 20 studies.^41^ It appeared that neither surgical intervention (OR 1.01, 95% CI 0.40-2.59) nor neurological deficit on presentation (OR 0.88, 95% CI 0.40-1.92) were significant variables in predicting improved postoperative outcome.^41^

Strengths of our study include the clear lack of identification of any universal classification for primary pyogenic spinal infections. We introduce the hierarchical AO Spine Primary Infection Classification System and encourage future studies to clearly specify the medical or surgical intervention performed to avoid heterogeneous reporting. Limitations of this study include the methodology being a narrative rather than systematic review as well as the lack of high quality randomized controlled trials available. The predominance of retrospective studies susceptible to bias and confounding is at odds with the important and common nature of primary pyogenic spinal infections. It is also important to disclose the authors have been involved in the development of the AO Spine Primary Infection Classification System.

We propose for clinicians to adopt the AO Spine Primary Infection Classification System. Future studies should clearly report the indication for surgery and whether the procedure involved biopsy, drainage, decompression, debridement, stabilization, reconstruction, or treatment of associated collections. In addition, it is imperative there is documentation of the antimicrobial agent, route, and duration which should account for important differences in antibiotic treatment between the surgical and medical cohorts. This is to act as a true control. Further prospective multi-centre validation would granulate the status of the AO Spine Primary Infection Classification System as a robust means of stratifying the severity of primary pyogenic spinal infection.

## Conclusion

Patients who suffer from pyogenic primary spinal infections present at different time courses along their illness and therefore existing studies examining whether early or late surgical intervention improves outcomes are investigating a heterogeneous cohort of patients. Existing studies remain disparate and flawed due to a lack of standardization and the absence of a reliable classification until now. Future research should be reported in a standardized fashion. The AO Spine Primary Infection Classification System represents a promising universal and logical means of communication which still requires prospective validation before its use in future management algorithms.
